# Editorial: Insights in pediatric neurology: 2021

**DOI:** 10.3389/fneur.2022.1041204

**Published:** 2022-11-01

**Authors:** Hong Ni, Pasquale Striano, Jo M. Wilmshurst

**Affiliations:** ^1^Division of Brain Science, Institute of Pediatric Research, Children's Hospital of Soochow University, Suzhou, China; ^2^IRCCS “Giannina Gaslini”, Genova, Italy; ^3^Department of Neurosciences, Rehabilitation, Ophthalmology, Genetics, Maternal and Child Health, University of Genova, Genova, Italy; ^4^Paediatric Neurology Division, Department of Paediatrics and Child Health, Red Cross War Memorial Children's Hospital, Neuroscience Institute, University of Cape Town, Cape Town, South Africa

**Keywords:** pediatric neurology, multiple center clinical trial, CDKL5, COVID Infection, epilepsy

Over the past few years, scientists have made exceptional achievements, resulting in major advancements in the rapidly evolving field of pediatric neurology. This Research Topic contains 19 articles (including 15 original research articles, one brief research report, two case reports, and one review), with contributions from 209 authors from 13 countries. The theme focuses on the most recent discoveries, latest advances, ongoing challenges, and future perspectives in the field of pediatric neurology. Diverse topics are covered inclusive of the international consensus recommendations for CDKL5 deficiency disorder, and the neurological and psychological presentation in children, and young populations with COVID Infection. The collection includes new findings and scales in pediatric psychiatry and psychotherapy and the diagnosis and intervention of different pediatric neurological disorders.

Multinational and multi-center cooperative research is the highlight of this topic. The CDKL5 kinase gene encoding mutations rank amongst the most common genetic childhood epilepsies and can manifest as the severe neurodevelopmental condition CDD (*CDKL5* deficiency disorder) (1). One contribution to this Research Topic by Amin et al. involved 18 pediatric medical and research institutions from the United Kingdom, Spain, France, the United States of America, Australia, and Italy, aiming to provide international expert consensus on recommendations for *CDKL5* Deficiency Disorder that will aid approaches to standardize and improve care for individuals with CDD. As a rare subacute complication of intrathecal or high-dose Methotrexate (MTX) administration stroke-like syndrome (SLS) can occur. Santangelo et al. from 12 Italian clinical and research institutes retrospectively described patients diagnosed with SLS at four major referral centers for Pediatric Hematology-Oncology. The results supported previous findings but in addition, found a linear correlation between age and disease severity.

Epilepsy is one of the most common pediatric neurological disorders. The antiseizure effect of the ketogenic diet (KD) is the focus of recent pediatric neuroclinical and basic fields (2–7), but there is still a need for multicenter studies in other pediatric neurological diseases.

Fang et al. reported on children with tuberous sclerosis complex (TSC) with drug-resistant epilepsy (DRE) and cognitive impairment, from 10 major city hospitals in China, for the efficacy and safety of KD to manage these co-morbidities. They found that KD could reduce seizure frequency and potentially improve cognition and behavior for this group. Yuan et al. analyzed the interictal discharges (IID) from pre-operative surface-electrode electroencephalograms (EEG) and compared the IID pattern changes post surgical excision of epileptogenic tubers in preschool children with TSC-related epilepsy. Those with post-operative seizure freedom were more likely to have non-IIDs vs. those with new focal IIDs were less likely to have seizure freedom at 3-year follow-up. Baker et al. investigated patients with infantile spasms (IS) for their longitudinal health outcomes post prednisolone (PRED) compared to Adrenocorticotropic Hormone (ACTH) treatment characterized using a phenome-wide association study. The findings were similar, across neurological and non-neurological outcomes. In addition, Makridis et al. retrospectively evaluated the outcome of 16 children with epilepsy who were treated with cenobamate, a drug for the treatment of adults with focal-onset epilepsy. The agent was effective and well-tolerated, suggesting that it could be a novel treatment for pediatric patients.

Yu et al. presented a Chinese patient with mild developmental delay who was found to have a *de novo* truncating variation in *SATB1*. Their study enhances the knowledge gap on the prognosis and treatment of rare neurological developmental disorders caused by gene mutations.

The current series on the neurological impact of COVID-19 on children has focused on severe multisystem inflammatory syndrome (MIS-C) with neurologic symptoms or other rare neurologic sequelae. Riva et al. describe a large group of children who had been infected by COVID-19, discussing their neurological complications and investigating these findings in relation to disease severity and population demographics. Except for headaches, the group found that neurological manifestations are an unusual presenting feature, and disease severity was not related to the pre-existing medical state. Guido et al. assessed the long-term outcome psychological consequences of COVID-19 infection on children and found that some symptoms were still present 3–5 months after infection. The data demonstrate that long-COVID presents psychological and ongoing cognitive issues, requiring intervention to avoid compromise on the quality of life of children and adolescents.

Learning and memory impairments have been the focus of pediatric neuropsychiatry and psychology due to the association between reading achievement and socioeconomic status (8, 9). An exploration of brain volume in patients with dyslexia by Ligges et al. found that reading deficits in those affected have gray matter volume variances in the reading network compared to unaffected readers. Behavioral improvement in reading skills was identified in different brain anatomical patterns, supporting the notion that dyslexia has lifelong consequences requiring consistent support in educational and professional career pathways. Stubberud et al. demonstrated that following pediatric acquired brain injury (pABI), diverse medical factors are associated with functional school outcomes. The study supported reintroduction to school with personalized programs tailored to the child's specific needs. Early identification and intervention of children at risk of learning disorders (LD) may improve outcomes. This tool is lacking in mainland China. The Preschool Learning Skills Scale was adapted by Yao et al., who created a Chinese version. This adapted version was then investigated for its validity and reliability and found to have good reliability and validity. In addition, a review by Melillo et al. explores the debate on whether Autism Spectrum Disorder (ASD) may be related to interregional brain functional disconnectivity as part of maturational delays in brain networks, in particular, the role of the inhibition of retained primitive reflexes (RPRs).

The early predictive value of novel parameters and markers in the diagnosis and prognosis of childhood neurological diseases has also been a key area of research in recent years (10). In pediatric Guillain-Barré syndrome (GBS) Jin et al. explored cerebrospinal fluid neurofilament light chain (CSF-NfL) levels as a potential prognostic biomarker and found that high CSF-NfL levels predict worse motor function and poor short-term prognosis of pediatric GBS. Pizzo et al. evaluated the incidence and prognostic value of brain MRI lesions and increased cerebrospinal fluid protein in children with Guillain-Barré syndrome. The results suggest a correlation between the MRI score, CSF protein, and prognosis. Du et al. retrospectively analyzed manifestations of cerebral paragonimiasis in children through neuroimaging. They found that lesions were mostly located in the cerebral parenchyma plus involved adjacent meninges, which could be of diagnostic value. Ma et al. found that independent ambulation as a milestone combined with the reading-frame rule significantly improved the early diagnosis of Duchenne muscular dystrophy (DMD).

The application of new technologies and methods in clinical diagnosis and treatment is also reflected in this special topic. Liu et al. investigated novel biomarkers and mechanisms related to Friedreich's ataxia (FRDA) progression. The results demonstrated that *CD28, FAS*, and *IFIT5* downregulation may be associated with disease progression. For patients with FRDA, pathogenesis may be related to the RNA regulatory pathway driven by NEAT1-hsa-miR-24-3p-CD28. Vališ et al. using the Czech National Registry for multiple sclerosis reported on affected children treated with disease-modifying drugs in 2013–2020.

In conclusion, the present clinical studies shed light on progress in Pediatric Neurology and its future challenges. We hope that the information gathered from this Research Topic will inspire, update and provide guidance to researchers in the field.

## Author contributions

JW is the leader of the Research Topic. HN wrote the draft. JW and PS reviewed the manuscript. All authors contributed to the article and approved the submitted version.

## Conflict of interest

The authors declare that the research was conducted in the absence of any commercial or financial relationships that could be construed as a potential conflict of interest.

## Publisher's note

All claims expressed in this article are solely those of the authors and do not necessarily represent those of their affiliated organizations, or those of the publisher, the editors and the reviewers. Any product that may be evaluated in this article, or claim that may be made by its manufacturer, is not guaranteed or endorsed by the publisher.

## References

[1] Khanam T Muñoz I Weiland F Carroll T Morgan M Borsos BN. CDKL5 kinase controls transcription-coupled responses to DNA damage. EMBO J. (2021) 40:e108271. 10.15252/embj.2021108271 34605059 PMC8634139

[2] Samia P Naanyu V Cross JH Idro R Boon P Wilmshurst J. Qualitative exploration of feasibility and acceptability of the modified Atkins diet therapy for children with drug resistant epilepsy in Kenya. Epilepsy Behav. (2021) 125:108362. 10.1016/j.yebeh.2021.108362 34740092

[3] Ni H Zhao DJ Tian T. Ketogenic diet change cPLA2/clusterin and autophagy related gene expression and correlate with cognitive deficits and hippocampal MFs sprouting following neonatal seizures. Epilepsy Res. (2016) 120:13–8. 10.1016/j.eplepsyres.2015.11.021 26709877

[4] Tian T Li LL Zhang SQ Ni H. Long-term effects of ketogenic diet on subsequent seizure-induced brain injury during early adulthood: relationship of seizure thresholds to zinc transporter-related gene expressions. Biol Trace Elem Res. (2016) 174:369–76. 10.1007/s12011-016-0730-3 27147436

[5] Zheng YQ Jin MF Suo GH Wu YJ Sun YX Ni H. Proteomics for studying the effects of ketogenic diet against lithium chloride/pilocarpine induced epilepsy in rats. Front Neurosci. (2020) 14:562853. 10.3389/fnins.2020.562853 33132826 PMC7550537

[6] Marchiò M Roli L Lucchi C Costa AM Borghi M Iughetti L. Ghrelin plasma levels after 1 year of ketogenic diet in children with refractory epilepsy. Front Nutr. (2019) 6:112. 10.3389/fnut.2019.00112 31396519 PMC6668051

[7] Ling Y Wang DD Sun YX Zhao DJ Ni H. Neuro-behavioral status and the hippocampal expression of metabolic associated genes in wild-type rat following a ketogenic diet. Front Neurol. (2019) 10:65. 10.3389/fneur.2019.00065 30804881 PMC6370680

[8] Colé P Cavalli E Duncan LG Theurel A Gentaz E Sprenger-Charolles L. What is the influence of morphological knowledge in the early stages of reading acquisition among low SES children? A graphical modeling approach. Front Psychol. (2018)9:547. 10.3389/fpsyg.2018.00547 29725313 PMC5917267

[9] Duncan LG Seymour PH. Socio-economic differences in foundation-level literacy. Br J Psychol. (2000) 91(Pt 2):145–66. 10.1348/000712600161736 10832511

[10] Chen JR Jin MF Tang L Liu YY Ni H. Acute phase serum leptin, adiponectin, interleukin-6, and visfatin are altered in Chinese children with febrile seizures: a cross-sectional study. Front Endocrinol. (2020)11:531. 10.3389/fendo.2020.00531 33042001 PMC7522506

